# Spectral interferometric microscopy reveals absorption by individual optical nanoantennas from extinction phase

**DOI:** 10.1038/ncomms4748

**Published:** 2014-04-30

**Authors:** Sylvain D. Gennaro, Yannick Sonnefraud, Niels Verellen, Pol Van Dorpe, Victor V. Moshchalkov, Stefan A. Maier, Rupert F. Oulton

**Affiliations:** 1 Department of Physics, The Blackett Laboratory, Imperial College London, London SW7 2AZ, UK; 2 INPAC, K. U. Leuven Celestijnenlaan 200 D, Leuven B-3001, Belgium; 3 IMEC, Kapeldreef 75, Leuven 3001, Belgium

## Abstract

Optical antennas transform light from freely propagating waves into highly localized excitations that interact strongly with matter. Unlike their radio frequency counterparts, optical antennas are nanoscopic and high frequency, making amplitude and phase measurements challenging and leaving some information hidden. Here we report a novel spectral interferometric microscopy technique to expose the amplitude and phase response of individual optical antennas across an octave of the visible to near-infrared spectrum. Although it is a far-field technique, we show that knowledge of the extinction phase allows quantitative estimation of nanoantenna absorption, which is a near-field quantity. To verify our method we characterize gold ring-disk dimers exhibiting Fano interference. Our results reveal that Fano interference only cancels a bright mode’s scattering, leaving residual extinction dominated by absorption. Spectral interference microscopy has the potential for real-time and single-shot phase and amplitude investigations of isolated quantum and classical antennas with applications across the physical and life sciences.

## Abstract (web summary)

Absorption by an optical nanoantenna determines its interaction strength with light, yet this quantity is hidden from conventional spectroscopy. Gennaro *et al*. now demonstrate a spectroscopic technique that reveals a nanoantenna’s absorption by recovering its amplitude and phase response.

## Graphical abstract

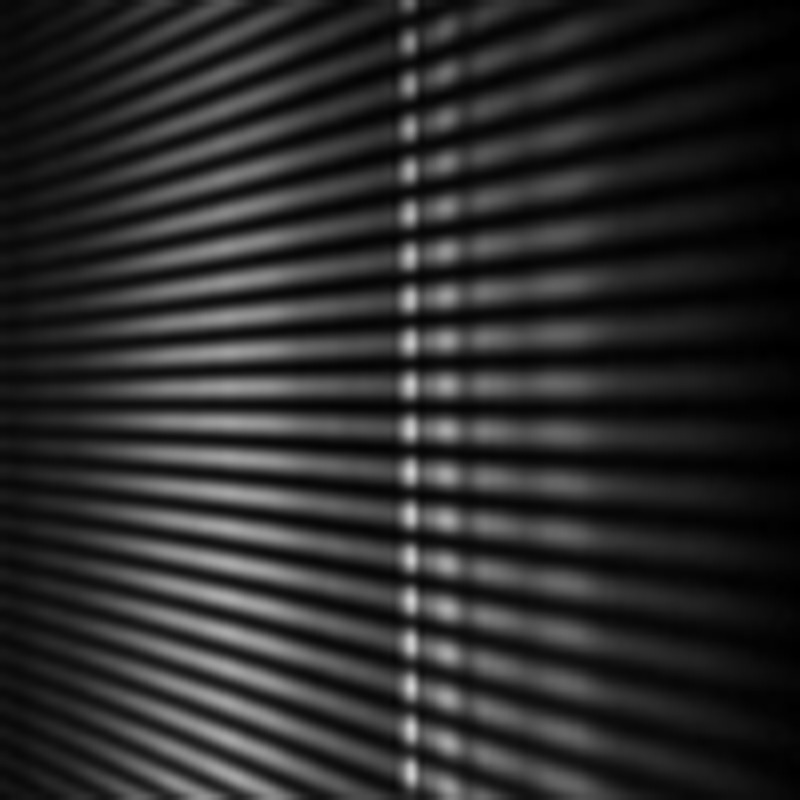

Nanoscale metallic particles and ‘molecules’ are now routinely used as optical frequency antennas^1^,^2^,^3^. Electrons at the surface of metallic nanostructures interact strongly with light via surface plasmon resonances that are tuneable simply by engineering the shape and size of the particle, leading to a myriad of different applications. Collectively, arrays of relatively large antennas can mimic the behaviour of atoms in solids, but with a much stronger interaction with free-space beams, providing flexibility to engineer the effective macroscopic permittivity and permeability^4^,^5^. Arrays of antennas are also used for light-harvesting photovoltaics^6^ and information storage^7^. Individually, small antennas strongly localize light at the nanoscale, which can be exploited to accelerate the electronic transitions in nearby materials^8^,^9^, leading to improved sensing^10^,^11^, enhancement of naturally weak nonlinear effects^12^,^13^ and near-field imaging^14^,^15^. Recently, a number of groups have devised methods of extracting both phase and amplitude of the scattering properties of metallic particles. In the field of metamaterials, the phase change through one or more layers of a collection of antennas is a direct and unambiguous measure of the refractive index^16^,^17^. Interferometry has also been used to study individual nanoparticles, for example, to detect sub-10 nm particles through local heating perturbations^18^,^19^; in nonlinear pump probe studies of surface plasmon dephasing processes^20^; and more recently for distinguishing proportions of scattering and absorption from the total extinction^21^. Such interferometry techniques are also relevant beyond metal optics; for example, the proposed perfect reflection of light from infinitesimal dipoles^22^ and the associated implications in nonlinear quantum optics^23^,^24^.

In this paper, we show how far-field amplitude and phase information allows quantitative estimation of a nanoantenna’s absorption, a near-field quantity. The absorption is an important parameter of any nanoantenna because it relates directly to optical intensity in the near field^25^,^26^. This tells us how efficiently an antenna can store electromagnetic energy at the nanoscale and therefore how strongly light–matter interactions may be enhanced^3^. Conventional spectroscopy only provides access to amplitude information, which essentially informs us of the total extinction of a nanoantenna; the underlying contributions of scattering and absorption are inaccessible. Nonetheless, nanoantennas always scatter light with a small phase shift that can directly be measured in the far field. Techniques capable of accessing the transmission amplitude and phase of a nanoantenna could thus measure both extinction and scattering providing all the necessary information to determine absorption. There is a critical technical challenge to determining the absorption of optical nanoantennas in this way. Surface plasmons evolve on time scales comparable to the collision time of electrons in metals (~10 fs), leading to broad extinction spectra with underlying phase changes less than the maximum value of *π*. Moreover, since the scattering strength is strongly dependent on a nanoantenna’s size and geometry, detectable phase changes in most cases requires focussed beam excitation; for example, the work of Zumofen *et al*.^22^ shows how focussed beam excitation can induce near perfect reflection even from infinitesimal dipoles. The technical challenge is therefore to integrate spectroscopy, interferometry and microscopy, with the principal difficulty being the dispersion of microscope glasses that induce group delay dispersion far exceeding what we can expect from broadband nanoantennas.

## Results

### Spectral interferometry microscopy

**Figure 1:**
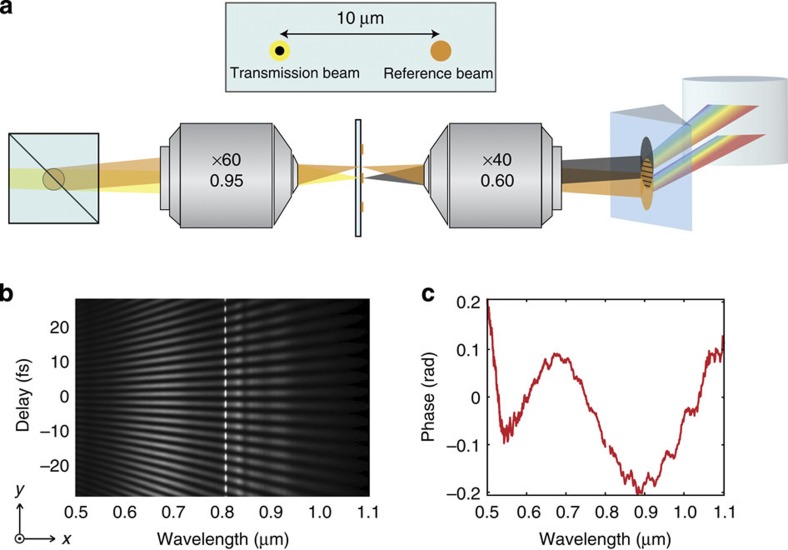
Illustration of the SIM technique. (**a**) Schematic of SIM. A tilted beam splitter introduces a vertical angle between two beam paths. Both beams propagate through the same microscope objective, appearing as two different focal spots separated by about 10 μm on the sample, one of which passes through the nanoparticle’s position. After the second objective, a prism and a cylindrical lens disperse the light onto a CCD camera. (**b**) An interferogram of wavelength (*x* axis) versus delay (*y* axis). (**c**) The system phase without a nanoparticle. The common path arrangement cancels most of the system’s group delay dispersion.

Spectral interferometry (SI) is an established technique that integrates spectroscopy with interferometry to access phase and amplitude information of light. SI resolves interference spectra of two optical signals as a function of their time delay. While older SI implementations employed delay lines and collinear beams to construct time-frequency maps, spatially encoded arrangements have simplified the technique and eliminated the time-frequency sampling limitation^27^,^28^. Here, temporal scanning is achieved by interference of the two signals propagating with a very small relative angle, creating spatial fringes that can be imaged by a camera to produce a two-dimensional (2D) frequency (*x* axis) versus delay interferogram (*y* axis)^29^. An example interferogram is presented in Fig. 1b. When integrated within our imaging system, SI microscopy (SIM) passes both signal and reference beams through the microscope converting the small relative angle between beams into two distinct spots in the focal plane, allowing single nanoantenna interferometry in a common path arrangement (see Methods). The SIM setup is shown in Fig. 1a. Unlike typical common path interferometers^21^,^30^, which use polarization to distinguish beam paths (for example, by using a pair of Wollaston prisms), the finely tuneable relative path angle makes SIM a polarization insensitive technique, providing access to the full scattering matrix of a general nanostructure; for example, the ring-disk dimer explored in this work^31^. Moreover, since the two beams follow a similar path through the same optical system, SIM manages the strong dispersion of the microscope glasses that would occur if only one of the interferometer’s beams had passed through the microscope. This enables us to eliminate any residual group delay dispersion of the imaging system, leaving only fourth order dispersion terms spanning just 0.3 radians, which is consistent with the apochromatic correction of the microscopy system (Fig. 1c). (See Methods for phase retrieval algorithm.)

### Absorption retrieval from transmission phase

**Figure 2:**
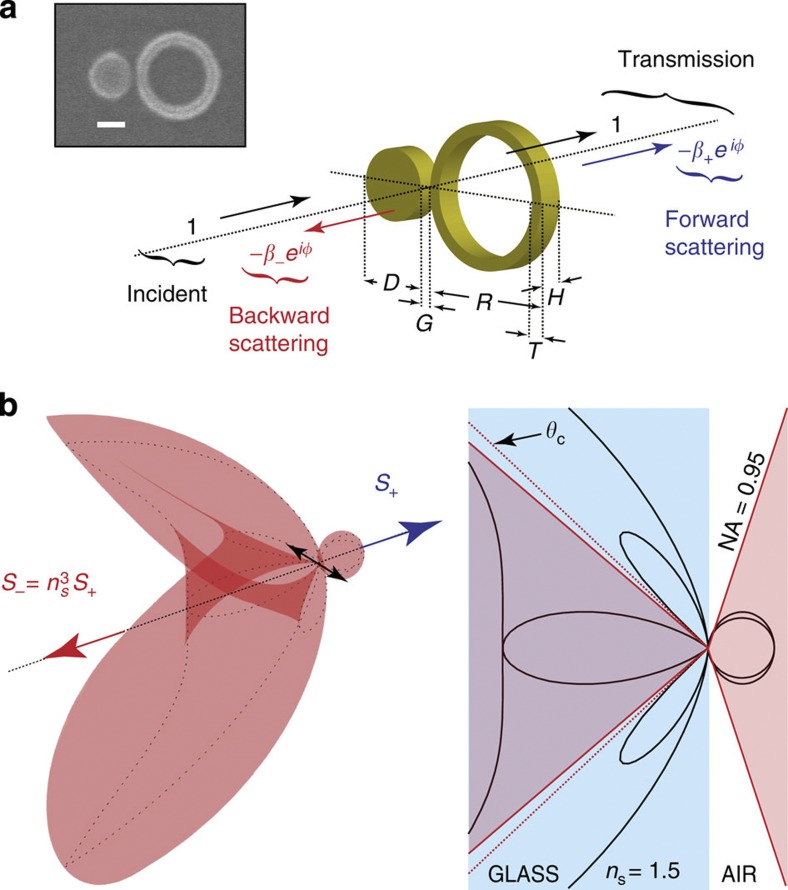
Illustration of an optical antenna scattering. (**a**) The structural dimensions of the plasmonic gold dimer studied are: *D*=230 nm, *R*=400 nm, *T*=60 nm, *G*=10 nm and *H*=60 nm. A SEM picture of the particle is shown in inset. (Scale bar, 200 nm.) Transmission is a sum of the forward scattered field and the incident beam. (**b**) Radiation pattern of a dipole on a glass–air interface. The substrate modifies the radiation pattern to resemble a Lambertian. Please note that the nanoantenna is illuminated from the glass side so that the high NA of the incident beam envelops most of the forward scattering distribution.

SIM enables the study of absorption and scattering processes of isolated nanoparticles by accessing phase information in transmission. Recent work has used the optical theorem^21^ combined with interferometric measurements to determine absorption and scattering components to extinction. Under plane wave (or weakly focussed) illumination, power flows along the optical axis, and consequently, the reduction in transmission occurs only along this axis; the optical theorem then relates the forward scattering amplitude along the optical axis to the total extinction cross-section. Critically, this assumes uniform dipole-like scattering of the nanoantenna. Under focused beam illumination, power flows along many directions, and so interference generally depends on the shapes of dipole and incident radiation^22^ and the optical theorem cannot be used. Under general illumination conditions, the transmitted electric field in the far field, **E**_***T***_(**r**, *ω*), is the interference of the illumination beam, **E**_**0**_(**r**, *ω*), and scattered, **E**_***S***_(**r**, *ω*), electric fields, also in the far field. An analytical description of the problem is forthcoming under the Born approximation^25^ where we assume that the scattered field is related to the incident field^32^ at the position, **r**_**0**_, of a point scatterer, 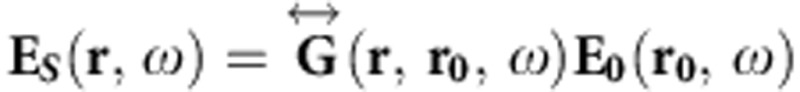, where 
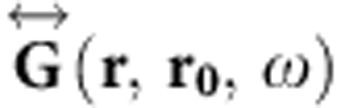
 describes the shape and polarization of the scattering, such that (Fig. 2a)


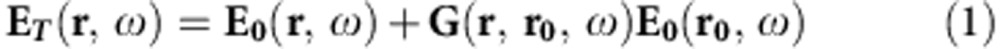


The transmission of the incident beam is given by a spatial overlap integral, 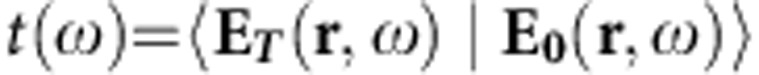, where we assume that 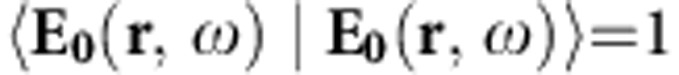 and 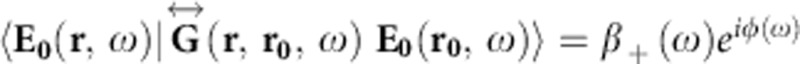 are the scattering amplitudes in the forward direction such that,


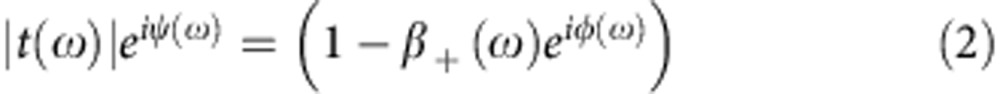


where |*t*(*ω*)| is the transmission amplitude and 
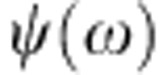
 is the transmission phase. The negative sign of the scattering amplitude, −*β*_+_(*ω*)*e*^*iφ*(*ω*)^, ensures that interference can only be destructive to conserve energy. The reader will note that *β*_+_(*ω*)*e*^*iφ*(*ω*)^ is the proportion of the scattering that coherently interferes with the incident beam in the forward direction. It does not include scattered light outside of the focused beam’s numerical aperture (NA) as shown in Fig. 2b. Moreover, in theory, any mismatch between the shape of the focussed beam and the scatterer’s radiation pattern^22^ reduces the scattering amplitude. This additional factor to the extinction is complicated by the substrate, which modifies the scatterer’s radiation pattern (Fig. 2b). In the forward direction, the scattering distribution of a dipole on a substrate now resembles a Lambertian providing an excellent match between the shapes of the focussed beam and dipole radiation pattern, and so we assume that all forward scattered light interferes with the incident beam. The total forward scattered power is therefore, 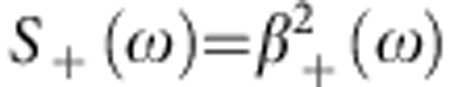.

The substrate also affects the total backscattered power, *S*_−_(*ω*). As can be seen from the scattering pattern in Fig. 2b, the substrate radically modifies the shape of scattered light as well as the relative power into forward and backward directions. In this work, we are most concerned with the total backscattered power, which increases with the ratio of refractive indexes cubed^33^,^34^. This can be understood from the fact that the volume of *k*-space, which indicates the density optical states, also scales with the refractive index cubed. Therefore, we assume that 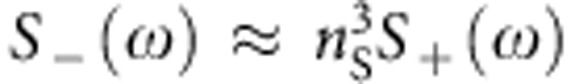, where *n*_S_ is the refractive index of the substrate. In this case, the absorption can be estimated to be,


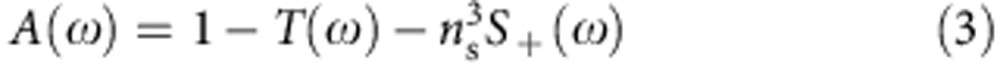


### Transmission amplitude and phase of a ring-disk dimer

**Figure 3:**
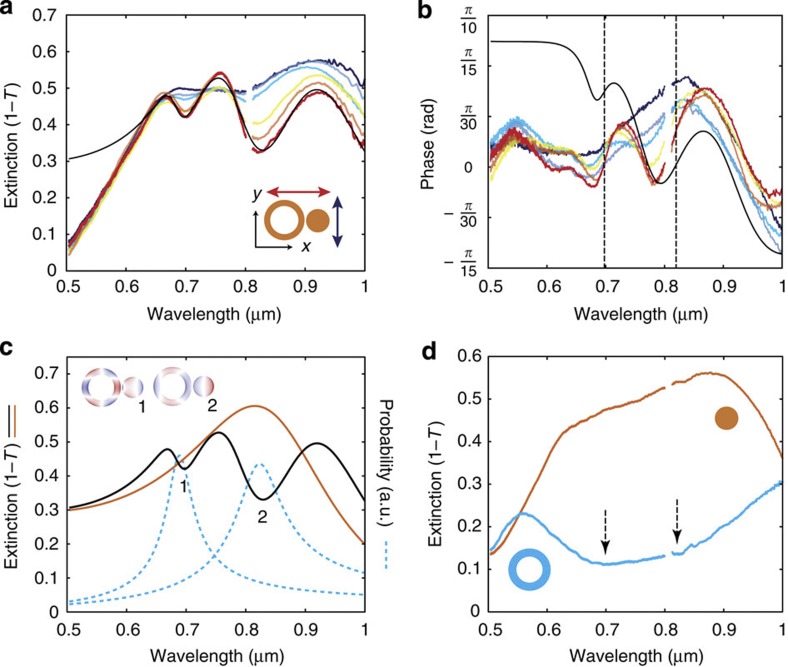
Extinction and transmission phase of an isolated ring-disk dimer. (**a**) Extinction of the ring-disk dimer for various excitation polarizations by 20° step (red curve is along the long axis of the dimer and dark blue curve is along the short axis of the dimer direction). The black line is a best fit of the oscillator model with one bright and two dark modes. (**b**) Transmission phase of the dimer for the various excitation polarizations by 20° step corresponding to the extinction spectra shown in **a**. The phase of the oscillator model fit in **a** is shown in black. The vertical black dotted lines indicate the location of the dark modes of the ring. (**c**) Response functions for the bright (orange) and dark (cyan) modes used in the oscillator model fit (black). The charge distributions of the structure at the Fano dips are shown in insets (See Methods). (**d**) Extinction for the individual ring and disk constituents of the dimer. The arrows indicate the location of the dark modes. Note that saturation of the spectral response near 810 nm due to the supercontinuum light source was removed.

We have used SIM to investigate a dimer consisting of a gold disk placed near a gold ring (Fig. 3d). The choice of this nanostructure is particularly relevant to show-case the SIM technique because it is known to exhibit Fano interference^31^,^35^,^36^, where near-field interactions influence far-field extinction. This is relevant because Fano interference is believed to result in the enhancement of near-field effects, such as enhanced second harmonic generation^26^,^37^. Discerning the absorption of an individual ring-disk dimer would provide access to near-field information that can directly explain these observations. External excitation of the ring-disk dimer couples to bright dipole-like modes, but the extinction is also influenced by internal dark modes of the ring due to its proximity to the disk^31^ causing a modulation of the extinction. Coupling to the internal dark modes of the ring can be controlled by the choice of polarization as shown in Fig. 3a. With an excitation polarization aligned with the long axis of the dimer (*x* axis), the disk dipolar extinction appears to exhibit Fano interference due to dark ring modes. Meanwhile, such resonances cannot be observed in the extinction spectra of either the individual disk or the ring, as shown in Fig. 3d. Figure 3a also shows that excitation polarization along the short axis of the dimer (*y* direction) does not lead to Fano interference. The extinction spectrum exhibiting the Fano interference (Fig. 3a) appears to agree reasonably well with a simple coupled oscillator model fit for one bright and two dark modes (see Methods). The responses of the bright and dark modes that constitute the modelled extinction are indicated in Fig. 3c, highlighting both their resonance positions and spectral widths. This agreement confirms the interpretation of Fano interference. However, the phase information from the SIM technique indicates a number of discrepancies that would be missed, as shown in Fig. 3b. The oscillator model agrees on the location of the dark modes but it overestimates the phase change for blue wavelengths, and therefore overestimates the proportion of scattering with respect to absorption. There are a number of possible reasons for this discrepancy: the strong absorption of gold below 520 nm is not included in the oscillator model; the bright mode of the ring near 550 nm is not included; and there appears to be a third dark mode near 620 nm, which is not apparent in the extinction, but quite clear in the phase measurement.

### Absorption and scattering measurements

**Figure 4:**
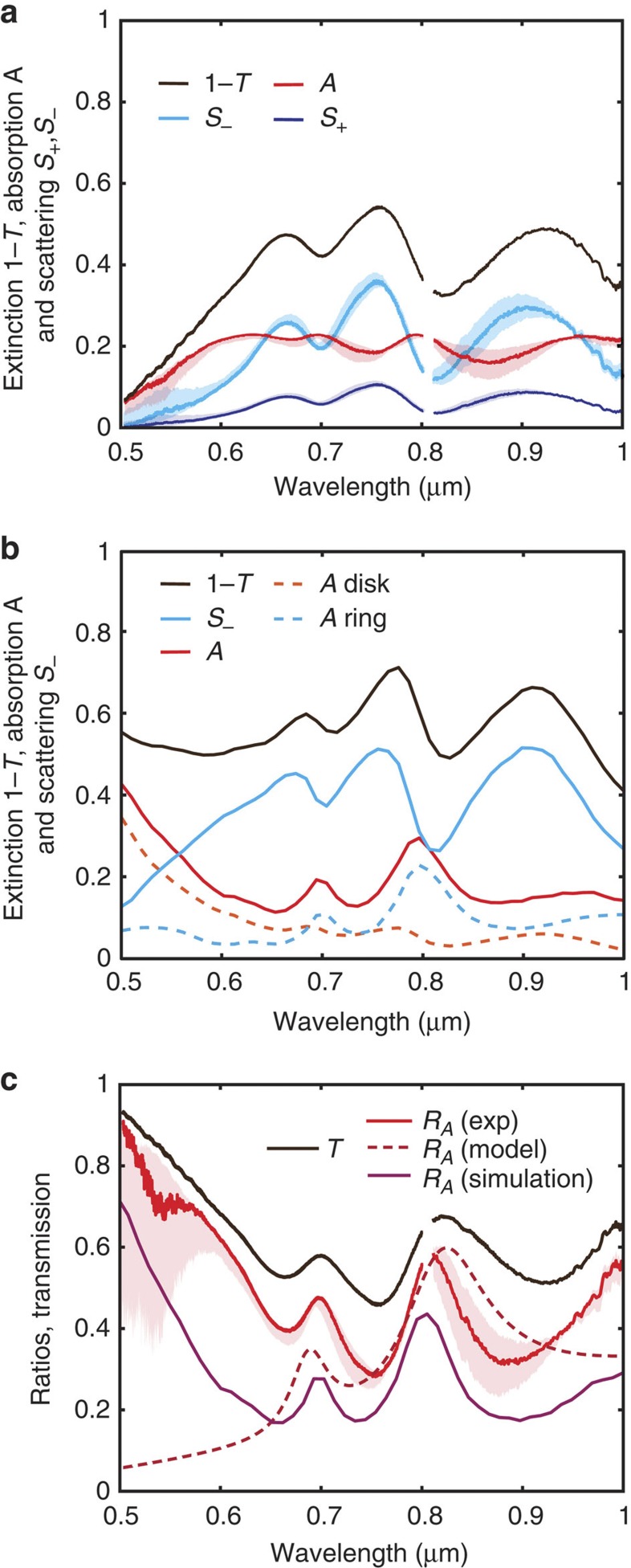
Comparison of the transmission, scattering and absorption of a ring-disk dimer. (**a**) A plot of transmission, forward scattering with the estimated backward scattering and absorption of the ring-disk dimer. (**b**) Simulation of the ring-disk dimer using Comsol: scattering, absorption and extinction. The absorption of the disk only and the ring only are also shown to illustrate that peaks in absorption indeed arise from the near-field excitation of the ring modes. (**c**) Comparison of the ring-disk dimer’s transmission with the ratio of absorption to total loss (that is, absorption, forward and backward scattering). This shows correspondence of the transmission peaks with the maximal proportions of absorption, supporting the observation of Fano interference. The shade areas represent the uncertainty due to the fluctuation of the beam paths (Supplementary Fig. 4).

The SIM technique can discern the proportions of scattering and absorption from the total extinction spectrum and the transmission phase using the procedure outlined earlier. Figure 4a shows the transmission, reflection (backward scattering) and absorption of the isolated ring-disk dimer. For completeness, Fig. 4a also shows the scattering in the forward direction. The most striking observation is the relative strengths of scattering and absorption across the spectrum. The absorption is relatively sustained while the scattering decreases at the wavelengths of Fano interference. This suggests that Fano interference cancels the bright mode’s scattering but introduces additional absorption, as the effect is mediated by inherently lossy dark modes of the ring. This is consistent with the Fano phenomenon, which provides an increase in transmission that can only be associated with a decrease in the amount of scattering from the bright modes of the structure^35^. Moreover, full-wave electromagnetic simulations confirm this result with remarkable correspondence between the observed trends in extinction, absorption and scattering (Fig. 4b, see Methods). Furthermore, the numerical simulations also confirm that the increased absorption at Fano interference arises directly from absorption in the ring and not the disk. Remarkably, these observations are not found in Fano-active nanoantenna arrays, which show a minimum in absorption at Fano interference^38^. This discrepancy could arise from the interference of radiation from nearby antennas in the far field, which would affect the balance of scattering and absorption at the Fano resonance. It is also pertinent to note that we clearly see that the proportion of absorption increases greatly nearer the band edge of gold near 520 nm.

The interplay of scattering and absorption during Fano interference can be examined further in Fig. 4c, which shows the ratio, *R*_*A*_(*ω*), of absorption compared with all scattering and loss processes of the dimer (that is, *R*_*A*_(*ω*)=*A*(*ω*)/(*A*(*ω*)+*S*_+_(*ω*)+*S*_−_(*ω*))). We clearly see that absorption dominates in the ring-disk system at Fano interference, which suggests an enhancement in the near-field response of the dimer. We can confirm this observation with the numerical simulations of the ring-disk system. Remarkably, the simulations predict the correct trend in the proportion of absorption relative to the total extinction, with only a slight shift in magnitude. The discrepancy is due to increased absorptive damping of the nanoantennas, most likely arising due to the conductive layer used for electron beam lithography (EBL) and the quality of the deposited gold. This also explains the higher extinction and scattering found in the numerical simulations (Fig. 4b). Clearly, experiments, numerical simulations and analytical theory are in agreement and confirm our hypothesis of the role of scattering and absorption in Fano interference. These findings shed light on the interplay of scattering and absorption and support SIM as a new tool for the engineering of optical nanoantennas to control enhanced light–matter interactions and scattering.

**Figure 5:**
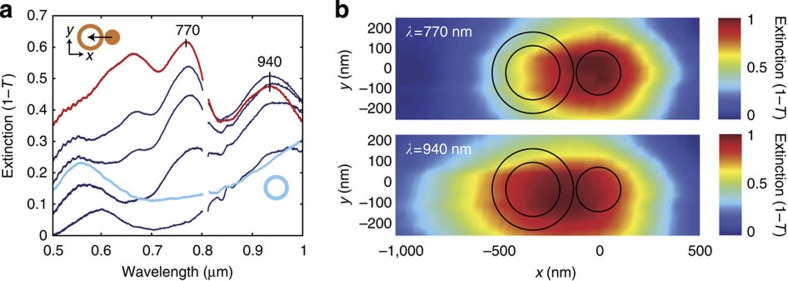
Spatially resolved extinction of an individual ring-disk dimer. (**a**) Extinction scans for the ring-disk dimer when the illumination spot is directed towards the ring. The particle is displaced relatively to the spot over a total distance of 1.5 μm in steps of 20 nm (see Methods). (**b**) *x*–*y* maps of the normalized far-field extinction at 770 nm and 940 nm of the ring-disk structure. We have assumed that the peak extinction at 770 nm only arises from the bright dipole mode of the disk (see Fig. 3d). The asymmetry of the plots (**a**,**b**) suggests that the dimer is oriented with the ring at negative displacements (Supplementary Fig. 1 and Supplementary Note 1).

Finally, it is also worth noting the utility of the microscopy element of SIM. Here we have scanned in 2D the dimer position relative to the focal spot measuring an extinction spectrum at each position (see Methods). The measurement in this case does not show us a near-field map, but a far-field extinction map of the structure. This allows us to confirm the spatial origin of Fano interference by accessing the distinct dipole modes of the disk and ring. Spectra taken along the *x* direction are shown in Fig. 5a. Since a focussed beam predominantly couples to dipole modes of the dimer, scanning of the beam relative to a particle’s position does not modify the spectral positions of the peaks and dips, but merely modifies their relative magnitude^39^,^40^ (Supplementary Fig. 1 and Supplementary Note 1). Following this approach, we can generate 2D extinction maps for certain wavelengths corresponding to the dipole modes of the disk only (*λ*=770 nm) and the combination of the disk and ring (*λ*=940 nm) as shown in Fig. 5b. The extinction maps are asymmetric along the *x* direction since the bright dipole mode of disk is spectrally distinct from those of the ring (Fig. 3d). The symmetric response along the *y* direction allows us to identify the correct orientation of the dimer in the experiment. The extinction map for *λ*=940 nm encompasses an asymmetric region of a similar size to the entire ring-disk dimer, indicating extinction arising from bright dipolar modes of both ring and disk, as expected. Meanwhile, the map for *λ*=770 nm has a smaller and shifted response that suggests extinction originates from just the disk. This confirms our interpretation of Fano interference of dark ring modes accessed through the dipolar mode of the disk.

## Discussion

We have demonstrated a novel method to extract absorption and scattering from phase and intensity measurements of extinction from individual optical nanoantennas by developing a spectrally resolved interferometer integrated within a confocal microscope, called SIM. Our technique bypasses the limitations of related approaches that rely on the optical theorem and polarization beam path control, making SIM broadly applicable to general nanoantennas. Most importantly, we have shown how to use the transmission phase of a nanoantenna under focussed beam illumination to quantitatively estimate the distinct contributions of scattering and absorption to the total extinction. We have demonstrated the capability of SIM by investigating a nanoparticle dimer exhibiting Fano interference. While we have highlighted the relative contribution of bright and dark modes in the overall extinction, the interferometry capability of SIM has revealed the distinct contributions of scattering and absorption from the dimer’s total extinction. Here we were able to observe the residual absorption during Fano interference, which is an inherently near-field effect. This capability of discerning scattering and absorption from the total extinction is extremely valuable for the entire gamut of applications exploiting metallic nanoantennas. With the underlying capability to image spectral and phase information and limited only by the aberrations and optical coatings of a relatively simple imaging system, SIM produces low-phase noise readout over a broad spectral range appropriate for studying both linear and nonlinear ultrafast scattering and absorption processes in nanoantennas.

## Methods

### Experimental arrangement of SIM and calibration

SIM is a spatially and spectrally resolving interferometer, enabling transmission amplitude and spectral phase measurements. It is classified as an amplitude division interferometer, as the amplitude of the wavefront is divided with a beam splitter, similar to the Bates wavefront interferometer introduced in the 1940s (ref. 41). Interference arises from a relative angle between two beam paths created by two non-polarizing broadband beam splitters in a Mach–Zehnder configuration, which introduces fringes in the region where both beams overlap. The first beam splitter creates two replicas of light from a supercontinuum fibre light source (femtowhite from NKT photonics pumped by nominally >150 fs pulses at 820 nm (Chameleon Ultra II)). One beam path is adjustable via a delay line so that both beams overlap in time and space at the cement layer of the second beam splitter. The second beam splitter’s adjustable orientation creates the small relative angle between beams (Fig. 1a). Both beams fill the back aperture of a × 60 0.95 NA Nikon Apochromat microscope objective. Due to the relative beam path angle, two spatially distinct focal spots occur. The particle under investigation is aligned with one focal spot, while the other is allowed to pass unhindered and serves as the reference for extracting a phase difference. The transmitted light is then collimated with a × 40 0.6 NA Nikon plan S Fluor microscope objective. A spectrometer, consisting of an F2 prism, a 100-mm cylindrical lens and a Pixelink CCD (charge-coupled device) Camera, images the resulting white light interferogram over wavelengths from 0.5 to 1 μm.

The common path arrangement of SIM has a low sensitivity to vibrations and high fringe visibility, as both beams experience similar dispersion and aberrations as they pass through the microscope. If only one beam went through the microscopy system, it would be difficult to compensate for high order dispersion and wavefront shape with an external optical system (see Supplementary Figs 2 and 3 as well as Supplementary Notes 2 and 3 for information on improving fringe visibility)^42^.

The setup is initially calibrated to determine the relative intensities of light through each interferometer path and the phase of the system. The intensity of each arm is measured by respectively blocking one arm of the interferometer and recording the spectra on the CCD camera. The phase of the system is extracted from the measured interferogram when both beams pass unhindered by nanoantennas. A nanoantenna is then placed in one of the beam paths to conduct the measurement of transmission amplitude and phase.

In imaging experiments, the particle is scanned by a closed-loop-controlled piezo stage within 5 nm accuracy. We initially recorded live extinction spectra and using computer control optimized the particle position to obtain the maximum peak extinction. Scans involved displacing the particle from this peak position in steps of 20 nm in the *x* direction and in steps of 50 nm in the *y* direction, which enabled us to create maps of the ring-disk dimer’s far-field extinction.

### Phase and amplitude numerical extraction algorithm

SIM measures the interference of the scattered and unscattered beam paths


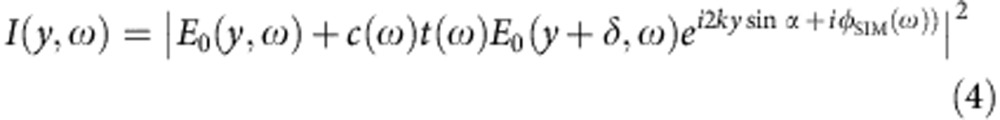


projected onto a CCD camera. The *y* axis represents the delay axis, but also incorporates the shape of the two beams shifted by a distance, *δ*, due to their relative incidence angle, *α.* 2*ky* sin *α* is the phase delay between the two beam paths, *φ*_SIM_ is the system phase and *c*(*ω*) is the ratio of amplitudes of the two beams. The *x* axis is produced by the dispersing prism and represents frequency. The phase is retrieved using 1D Fourier transforms along the vertical position, *y*, of the interferogram for each wavelength. The measured interference signal is a sum of constant and cosine terms. The Fourier transform in the frequency domain consists therefore of three bands: a central ‘DC’ band, which is the sum of the intensity of the two signals; and two sidebands on either side, which are interference terms^29^. One of the two sidebands is selected and Fourier transformed back to the spatial domain, providing the complex amplitude and relative phase,


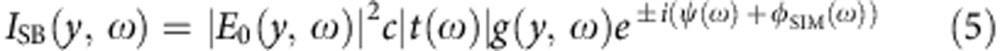


The choice of phase sign in the exponential arises from the selection of one of the sidebands. In practice, a beam overlap function, *g*(*y*, *ω*)=*E*_0_(*y*, *ω*)*E*_0_(*y*+*δ*, *ω*)/|*E*_0_(*y*, *ω*)|^2^, should only be a function of *y*; however, lens aberrations and the camera’s averaging effect over constantly fluctuating phase delay make it a weak function of *ω* (Supplementary Fig. 2). This does not influence the spectral phase, which can be extracted from the argument of the exponential. The phase is then averaged over each position, *y*, to reduce the impact of noise^43^,^44^. A Butterworth filter was used to reduce artefacts arising from the finite Fourier transform window. The accuracy of the phase retrieval depends on a number of experimental parameters: the relative beam angle, the fringe visibility and any lens aberrations. Small variations of the beam path between two subsequent measurements due to mechanical vibrations of the optical components and variations of ambient pressure and temperature can be corrected by removing the residual linear phase delay from a measurement (Supplementary Fig. 4 and Supplementary Note 4).

The crossing angle between the two beams is important for accurate phase retrieval, as it dictates the number of fringes on the interferogram. It should be large enough to separate the two sidebands from the central term, yet small enough to avoid pixel-fringe aliasing^28^. Imaging ~20 fringes seems to give the best result according to previous studies^45^, which corresponds to a full angle of *θ*≈0.004 radians on the camera of our imaging system. Note that the number of fringes on the camera is dependent on the magnification of the microscopy system. Care should also be taken to minimize the beams’ relative angle since the interference region where the two spots overlap reduces with distance from the second objective. Consequently, the second objective’s back focal aperture should be sufficiently larger to allow imaging of the interference zone.

### Oscillator model for nanoparticle scattering

The experimental transmission measurements for the nanoparticle dimer are compared with a coupled oscillator model, accounting for the various dark and bright modes of the system. These can be described by the Abraham–Lorentz equation^25^ with inter-particle coupling terms and driven by the incident electric field *E*_0_*e*^*iωt*^, such that


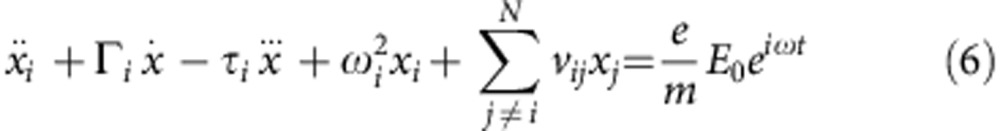


where Γ_*i*_ represents the non-radiative loss decay rate, *τ*_*i*_ represents the radiative loss decay time, *ω*_*i*_ the resonance frequency and *v*_*ij*_ are the coupling rates between oscillators *i* and *j.* Bright and dark modes are distinguished by the relative magnitudes of radiative and non-radiative rates.

The motion of the oscillator is linked to the scattered field by Lamor’s formula, 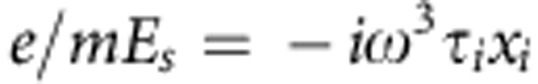 where *x*_*i*_ is found by solving the simultaneous equations above. The scattered field is also influenced by the substrate, which redistributes the total scattering power |*E*_*s*_|^2^ of the dipole between the forward 
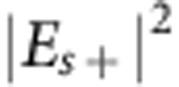
 and the backward scattering 
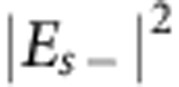
 accordingly to equations (7) and (8).


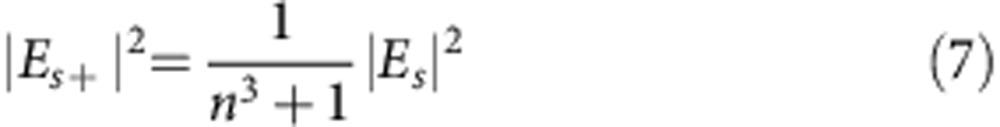



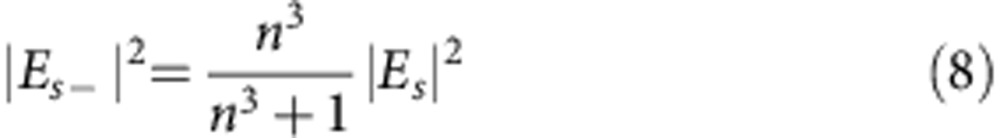


Where *n*_s_ is the substrate refractive index. In a simple 1D model, the interference of the scattered and incident fields gives us an expression for the complex transmission amplitude,


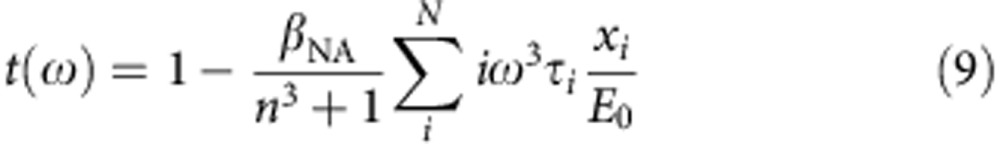


Here, *β*_NA_ accounts phenomenologically for the 3D mismatch in shape between dipole radiation and focussed beam fields, described earlier. In the model fit to the experimental data, the following parameters were used: (1) disk mode (2*πc*/*ω*_1_=821 nm, Γ_1_=0.202 fs^−1^, *τ*_1_^−1^=0.134 fs^−1^); (2) dark ring mode (2*πc*/*ω*_2_=822 nm, Γ_2_=0.201 fs^−1^, *τ*_2_^−1^=0 fs^−1^, *ν*_12_=0.9769, fs^−1^); and (3) dark ring mode (2*πc*/*ω*_3_=688 nm, Γ_3_=0.159 fs^−1^, *τ*_3_^−1^=0 fs^−1^, *ν*_13_=0.6615, fs^−1^).

### Ring-disk dimer numerical simulations

The simulations were conducted using Comsol (commercial finite element method solver) and Lumerical (commercial finite difference time domain solver). Results from both methods are found to be in general agreement; however, Comsol was more amenable to focussed beam excitation simulations. Extinction, scattering and absorption, used in Fig. 4, were calculated from the power flowing through the surfaces of calculation boxes surrounding the ring-disk structure in Comsol. Focussed beam simulations in Comsol assumed a Gaussian beam launched within the substrate with a beam waist, *w*_0_=*λ*/*nπθ*=*λ*/3.23, where *θ* is the beam divergence angle corresponding to NA=0.95, to match the experimental conditions. The charge distributions, shown in Fig. 3, were obtained by evaluating Poisson's equation from the complex fields extracted from the simulation in the vicinity of the top surface of the structure in Lumerical. The dielectric permittivity of gold is based on Johnson and Christy data^46^, and the refractive index of the substrate is *n*=1.5.

## Author contributions

S.D.G. and R.F.O. conceived of SIM, the method to extract absorption and scattering from extinction phase and implemented the experiments. Y.S., S.A.M., N.V., P.V.D. and V.V.M. designed and constructed the ring-disk nanoantennas. S.D.G., Y.S. and R.F.O. conducted the simulations. S.D.G., Y.S., S.A.M. and R.F.O. wrote the manuscript.

## Additional information

**How to cite this article:** Gennaro, S. D. *et al*. Spectral interferometric microscopy reveals absorption by individual optical nanoantennas from extinction phase. *Nat. Commun.* 5:3748 doi: 10.1038/ncomms4748 (2014).

## Supplementary Material

Supplementary Information Supplementary Figures 1-4, Supplementary Notes 1-4 and Supplementary References
