# Supplementary material for: Spectral interferometric microscopy reveals absorption by individual optical nanoantennas from extinction phase
Source: Nat Commun. 2014 Apr 30;5:3748. doi: 10.1038/ncomms4748 (PMC4015323; doi:10.1038/ncomms4748)
Supplement: Supplementary Information — Supplementary Figures 1-4, Supplementary Notes 1-4 and Supplementary References [file ncomms4748-s1.pdf]

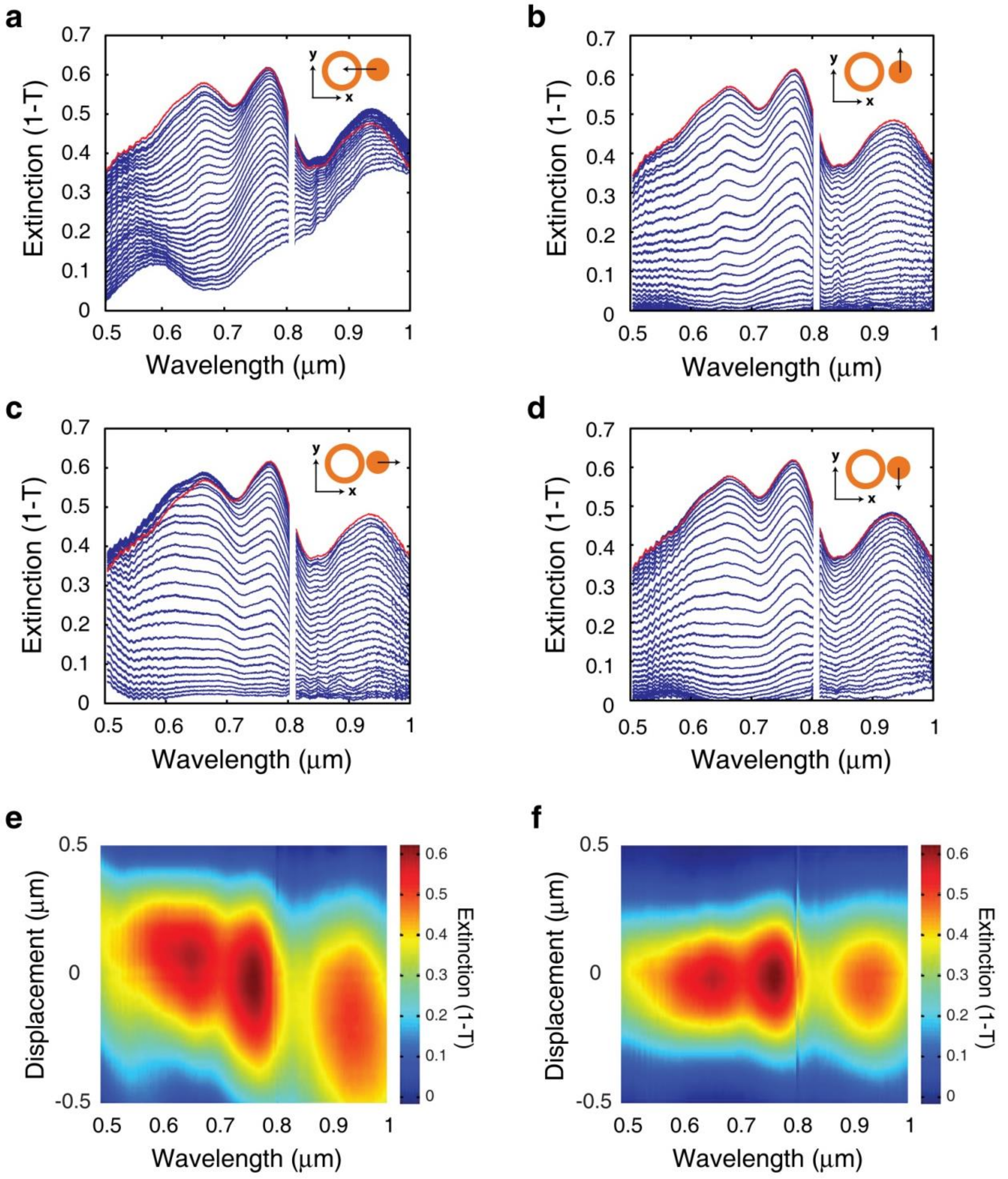

Supplementary Figure 1. Spatially resolved extinction of an individual ring-disk dimer. Linear scans of extinction when the spot is displaced along: **a** the x-axis toward the ring; **c** the x-axis away from the ring, and **b** & **d** the y-axis to either side of the particle. **e** and **f** show extinction maps versus position and wavelength. The particle is displaced over 1.5μm (1μm toward the ring and 0.5μm away from the ring) with steps of 20 nm. For clarity, only a displacement of 0.5μm toward the ring is shown in **a**&**e** to highlight the asymmetry of the response due to the presence of the ring. All the scans are with polarization along the x-direction of the Ring-Disk dimer. Note that saturation of the spectral response near 810nm due to the super-continuum light source was removed.

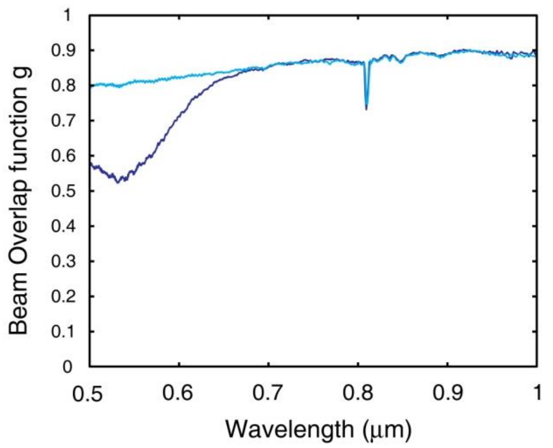

Supplementary Figure 2. Beam overlap function $g(y,\omega)$. The cyan line shows the beam overlap function of SIM. $g(y,\omega)$ **s**lightly decreases as a function of wavelength due to integration time of the CCD camera and chromatic aberrations of the optics. The blue line shows the reduction in the spectral overlap function of SIM beyond the operation range of an achromatic half wave plate used for polarization control.

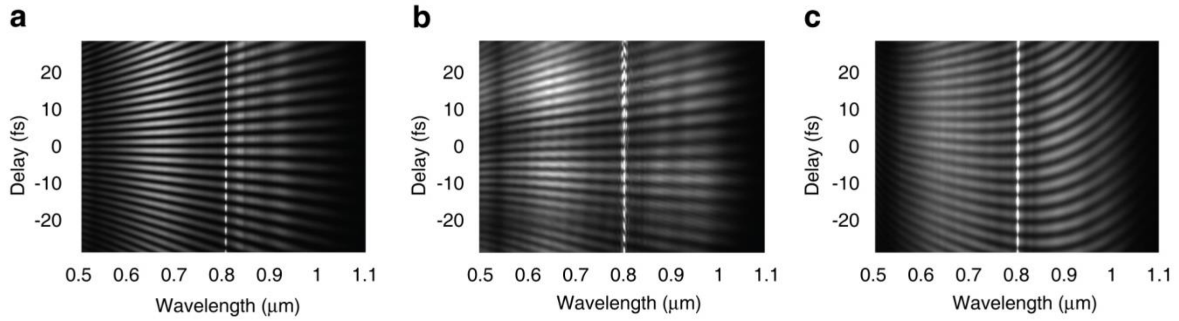

Supplementary Figure 3. Interferograms. **a** SIM approach **b** Reflective objective and **c** the interference beam passes through a dispersion compensating glass block instead of through the objective. The reflective objective has reduced visibility due to beam obstruction in the Cassegrain objective (Spherical and coma aberrations). Without the common path arrangement, the two beams will have different spatial and chromatic aberration leading to reduced visibility. The fringe curvature in this case is due to the remaining dispersion due to the different glasses used in each beam path.

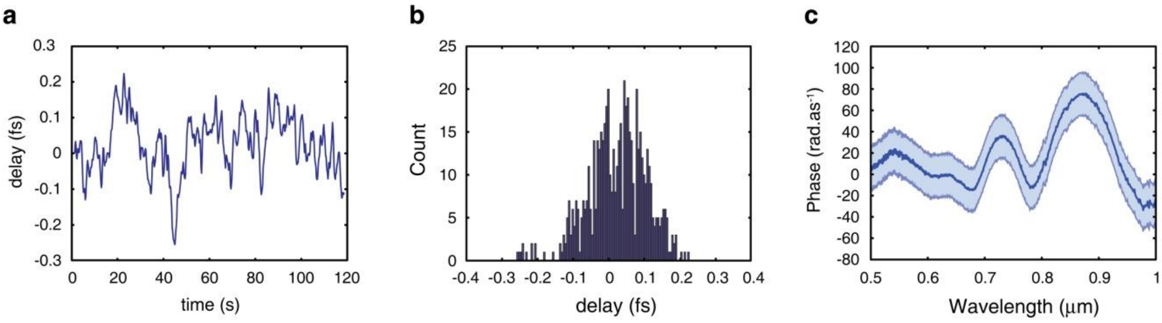

Supplementary Figure 4. Phase Noise delay. a Delay versus times. 600 frames have been recorded in total, i.e. 1 every 200 ms for 120 seconds. **b** Histogram distribution of the delay. The delay noise seems to be distributed from -0.1fs to 0.1fs. **c** Phase divided by the frequency change when a delay of -0.02fs and 0.02fs have been introduced to our best estimation of the zero phase (central dark blue line). Since the noise is a linear term, some known information about the phase and the short dynamic range enable to reduce the uncertainty of the delay range.

Supplementary Note 1 - Spatially resolved extinction of an individual ring-disk dimer

Since Spectral interferometry Microscopy (SIM) uses a confocal microscope it can also spatially resolve the extinction response of the metallic nanoparticle to give more information about how the structure is excited. In these experiments we first adjusted the particle position relative to the focal spot to optimize for peak extinction and then scanned along the x and y directions over a total range of 1.5 μm in steps of 20 nm.

Supplementary Figure 1 shows a series of scans of the extinction and two maps of extinction versus displacement and wavelength. When scanning away from the ring, Supplementary Figure 1b, c & d we can see the same extinction response shape that merely diminishes. Equally, in the case of the displacement along the y-axis, the map of the extinction versus displacement and wavelength (Supplementary Figure 1f) highlights the symmetry of the Fano dimer.

However, when scanning along the x-direction towards the ring, we observe a different extinction response that diminishes more slowly and eventually resembles the underlying ring's extinction spectrum, as shown in Supplementary Figure 1a. This is consistent with the focal spot being very near to the centre of the ring. This asymmetric response is highlighted in the map, in Supplementary Figure 1e. Since the ring is very large, the extinction is sustained for larger displacements.

These observations indicate that the optimum extinction occurs when the focal spot is located over the disk and supports the hypothesis of Fano interference of dark ring modes accessed through the dipolar mode of the disk.

Supplementary Note 2 - Beam overlap function at the CCD, $g(y, \omega)$

The interference fringes that Spectral Interferometry Microscopy (SIM) produces are imaged on a CCD camera. The fringe visibility is described by a beam overlap function, $g(y, \omega)$, which was defined in the Methods section of the main manuscript. Since SIM is an imaging spectrometer, the temporal coherence of each dispersed wavelength falling on a line of pixels is relatively high, so the interference visibility will persist for large enough delays to suggest that $g(y, \omega)$ should be independent of ω. However, in reality $g(y, \omega)$ depends on various factors: the spatial coherence of the light source[1], the integration time of the CCD camera[2], the polarization of the light, the optical correction of the microscope objective[3]and chromatic aberrations of the cylindrical lens of the spectrometer. Chromatic aberrations in specific can influence the spatial imaging of various wavelengths, and so makes $g(y, \omega)$ a weak function on frequency.

Supplementary Figure 2 shows the measured overlap function for the SIM set-up.

The overlap function depends on the light distribution of the two beams emanating from the light source. Assuming Gaussian beams of shape $|E_0(y, \omega)|^2 = f(\omega)\exp(-y^2/4\sigma^2)$, the overlap function as defined in the text is,

$$g(y, \omega) = \frac{E_0(y, \omega)E_0(y + \delta, \omega)}{|E_0(y, \omega)|^2} \approx 1 + \frac{\delta E_0(y, \omega)}{|E_0(y, \omega)|^2}\frac{dE_0(y, \omega)}{dy} = 1 - \frac{\delta y}{4\sigma^2}$$

Where $\delta = 2$ mm is the displacement of the two beams given their relative angle of 0.004 rad and $\sigma = 3.5$ mm is the beam width. When averaged of the whole camera of side $L = 3.57$ mm, we find

that the maximum average $\overline{g(\omega)} = 0.96$. Meanwhile the maximum value seen in experiments is just above 0.9. Spatial and chromatic aberrations in the microscope objective distort the shape of the wavefront, which reduces the spatial coherence of one wavefront relative to the other[3].

We should also note that the beam path is continuously changing and so the CCD camera integrates over many frames. Consequently, the interferogram recorded is an average over many frames and leads to slight blurring[2]. The random delay is a function of frequency due to the wavelength dependent refractive index of the various glasses of the optics and it is more dominant in the blue part of the spectrum, where we see a slight decrease of the visibility (Supplementary Figure 2). It is actually quite remarkably that such high quality interference fringes are observable, given these limitations.

Finally, care must be taken when using polarization optics in SIM. We placed a 690-1200 achromatic half wave plate in front of an uncoated Glan-Taylor polarizer. This enables us to rotate the polarization without shifting the position of the beam. However, the achromatic half wave plate only works properly from 690 up to 1200. Below, in the blue region, a portion of the light will be elliptically polarized. This seems to affect the beam overlap function, due to the effects of the elliptical polarization.

We have measured the beam overlap function by calculating the side band intensity, $I_{\pm}(\omega)$, and the DC intensity, $I_D(\omega)$, and comparing this with the intensity ratio of the two beams, c, as defined in the main text. It follows that,

$$\overline{g(\omega)} = \frac{2c(\omega)}{1 + c(\omega)} \frac{I_{\pm}(\omega)}{I_D(\omega)} \tag{1}$$

This measure of overlap function is less sensitive to the non-linearity of the CCD camera as the intensity ratio of the two beams $c(\omega)$ is insensitive and the side band intensity, $I_{\pm}(\omega)$, and the DC intensity, $I_D(\omega)$, are from the same interferogram.

Supplementary Note 3 - Fringe quality

Fringe visibility depends on the quality of the microscope objectives and minimizing the path difference between the two interfering beams. High visibility of the fringes leads to phase retrieval with lower noise[2]. Spectral interferometry visibility relies on the spatial coherence of the wavefront. Any that can change the shape of one wavefront relative to the other will reduce the visibility[3].

The visibility of the fringes depends on the spatial coherence of the light source, the integration time of the CCD camera, the optical correction of the microscope objective, the polarization of the light and chromatic aberration of the cylindrical lens, as described in section 'Beam overlap function at the CCD, $g(y,\omega)$'[3]. Supplementary Figure 3 shows some examples of interferograms from various methods tried in the development of SIM. The quality of fringes is clearly dependent on the optical arrangement used.

For the SIM interferogram, as shown in Supplementary Figure 3a, the common path arrangement allows both beams to pass through the microscope objectives. Both beam experiences almost similar optical aberrations so their relative shape does not change. Equally, as they travel through almost the same amount of glass, the spectral phase difference due to second order dispersion is low.

The second arrangement uses a reflective objective shown in Supplementary Figure 3b. The main features are the higher transmission in UV but some spherical and coma aberrations reduce the contrast of the fringes. The focus of the zero dispersion wavelength @800nm is not sharp and, since both beams travels at relative angle, the mirror inside the objective will obstruct different parts of the beam. This interferogram shows the importance of having high performance microscope objectives to obtain good quality fringes. However, with these microscope objectives, it is still possible to retrieve the phase with a good accuracy and low noise.

The last arrangement uses a separate path; one beam path goes through the microscope objective whereas the other travels through a glass block to compensate for dispersion. Supplementary Figure 3c shows the quality of the interferogram. The visibility is poor and the fringes appear to be bent with the wavelength as the imperfect correction of dispersion leaves some residual second and third order dispersion phase difference between the two arms. This may be eliminated but only to leave third order dispersion. Moreover, any optical aberration introduced by the microscope objective cannot be corrected with the glass block. Consequently, the wavefront shape is no longer a plane wave at the output of the second objective and the spatial coherence is reduced.

Supplementary Note 4 - Linear Phase Noise

SIM involves two interferometer paths (one as a reference and one as a probe). It can measure the relative phase difference between the two arms. In real time, the optical path difference continuously changes around its mean value, as shown in Supplementary Figure 4. These arise for three main reasons: mechanical vibrations of the optical elements of the interferometer, which causes nanometer changes in the optical path; air flow in the laboratory, which can cause optical elements to vibrate; and finally, temperature changes cause some optical components to dilate, which increase or reduce the path length of the interferometer[2].

Accessing only the phase shift induced by the particle on the laser beam with interferometry involves two measurements: one pre-measurement to remove the phase change of the system when both beams go through the microscope unhindered and another measurement when the particle is placed in one of the beams. During these two measurements, the optical path difference can vary, which causes a linear phase delay.

$$\phi_{System}(\omega) = \phi_{SIM}(\omega) + \tau_1\omega \tag{2}$$

$$\phi_{NP}(\omega) = \psi_{NP}(\omega) + \phi_{SIM}(\omega) + \tau_2\omega \tag{3}$$

Where $\phi_{SIM}(\omega)$ is the phase of the system, $\psi_{NP}(\omega)$ is the transmission phase of the particle and $(\tau_2 - \tau_1)\omega$ is the phase delay difference between two measurements, τ_1, τ_2 are the time delays at the two different measurements. Experimentally, we estimate this noise by recording 600 frames in total, i.e. 1 every 200 ms for 120 seconds. The short time scale means we can assume temperature equilibrium during each measurement, but not in between two different measurements.

To eliminate the system phase term, we subtract the phase of the reference interferogram so we can have access to the phase difference.

$$\phi_{NP}(\omega) - \phi_{System}(\omega) = \psi_{NP}(\omega) + \underbrace{(\tau_2 - \tau_1)\omega}_{Linear\ term} \qquad (4)$$

The linear delay can be exposed by simply dividing the measured phase result by the frequency. Supplementary Figure 4a and 4b show the variation of the random delay versus time and a histogram to show the statistics of the noise. It seems to be Gaussian noise central to zero with a slightly offset due to temperature drift in the lab.

Provided with a sufficiently broadband spectrometer, it is also possible to effectively use DC filtering methods to eliminate the random delay between various measurements. In our current work, the zero delay position is estimated using the information we already know about the phase. The spectral phase from scattering of a resonant particle has no constant or linear phase term. Additionally, it crosses zero near the peak wavelength of the resonance. In our case, this means we can estimate the zero in phase to be near the peak extinction of the bright disk mode. A pre-measurement of the resonance of the disk shows a peak wavelength between 0.9-0.95 µm. As the total phase is a combination of the phase of the disk modified with the presence of dark modes, we can estimate where the phase should cross zero. Supplementary Figure 4c shows the phase. By varying the delay, we can adjust the offset term. In the publication, we choose a variation of delay of ± 20 *as* around the optimum estimation of the phase at zero delay. This range represents our uncertainty in the linear phase delay. Its effect on the estimate of scattering and absorption can be seen in Figure 4 of the main text by the shaded areas around the phase dependent curves.

Supplementary References

1. Born, M. & Wolf, E. *Principles of optics*. (Pergamon Press, 1980).

2. Börzsönyi, A., Kovács, A., Görbe, M. & Osvay, K. Advances and limitations of phase dispersion measurement by spectrally and spatially resolved interferometry. *Opt. Commun.* **281,** 3051–3061 (2008).

3. Dyson, J. *Interferometry as a measuring tool*. (Machinery Publishing, 1970).
